# A retrospective study of treatment persistence and adherence to α-blocker plus antimuscarinic combination therapies, in men with LUTS/BPH in the Netherlands

**DOI:** 10.1186/s12894-017-0226-2

**Published:** 2017-05-22

**Authors:** Marcus J. Drake, Sally Bowditch, Emilio Arbe, Zalmai Hakimi, Florent Guelfucci, Ikbel Amri, Jameel Nazir

**Affiliations:** 1 Bristol Urological Institute and the School of Clinical Sciences, University of Bristol, Bristol, UK; 2 Astellas Pharma Europe Ltd, Chertsey, UK; 3 Astellas Pharma Europe B.V, Leiden, the Netherlands; 4 Creativ-Ceutical Ltd, London, UK; 5 Creativ-Ceutical Ltd, Tunis, Tunisia

**Keywords:** α-blocker, Antimuscarinic, Fixed-dose combination, LUTS/BPH, Treatment persistence

## Abstract

**Background:**

To assess treatment persistence and adherence in men ≥45 years of age with lower urinary tract symptoms (LUTS) associated with benign prostatic hyperplasia (BPH), using prescription records from the Netherlands IMS Lifelink™ LRx database.

**Methods:**

In this retrospective, observational cohort study, we identified men who received combination therapy with an α-blocker plus an antimuscarinic (e.g. solifenacin or tolterodine) between 1 November 2013 and 31 October 2014. Treatment could be received as a fixed-dose combination (FDC) tablet or as two drugs administered together (concomitant therapy), if both combination drugs were prescribed within 30 days. The primary objective was to assess treatment persistence, defined as the time from initiation of combination therapy until first discontinuation of the FDC or at least one of the drugs given concomitantly (i.e. ≥30 days without prescription renewal). Subgroup and sensitivity analyses were conducted to assess persistence by antimuscarinic agent, and with different gap lengths used to define discontinuation (45, 60 and 90 days), respectively.

**Results:**

A total of 1891 men received an α-blocker plus an antimuscarinic (FDC, *N =* 665; concomitant therapy, *N =* 1226). Median time to discontinuation was significantly longer with FDC versus concomitant therapy (414 vs. 112 days; adjusted hazard ratio [HR] 2.04, 95% confidence interval 1.77, 2.35; *p* < 0.0001). Persistence at 12 months (51.3% vs. 29.9%) was also significantly greater with FDC compared with concomitant therapy. Assessment of antimuscarinic subgroups showed that median time to discontinuation was longest with solifenacin combinations (214 days) compared with other antimuscarinic combinations (range, 47–164 days; adjusted HR range, 1.27–1.77, *p* = 0.037). No observable impact on treatment persistence was found by adjusting the gaps used to define discontinuation.

**Discussion:**

This study of real-world evidence of men with LUTS/BPH treated with α-blocker plus antimuscarinic combination therapy in the Netherlands showed that treatment persistence was significantly greater in those who received a FDC tablet compared with combination therapy given concomitantly. The study also shows that treatment persistence was extended in men who received combination therapy containing solifenacin compared with other antimuscarinics.

**Conclusions:**

Overall, these findings may be useful for prescribers, as improved persistence on-treatment may translate into improved outcomes for men with LUTS/BPH. Further study is warranted to establish the key drivers of persistence in men receiving combination therapy for LUTS/BPH.

**Electronic supplementary material:**

The online version of this article (doi:10.1186/s12894-017-0226-2) contains supplementary material, which is available to authorized users.

## Background

Lower urinary tract symptoms (LUTS) are most common in the ageing male population, with troublesome LUTS occurring in 30% of men over 65 years of age [1]. LUTS can be divided into voiding, storage and post-micturition symptoms [2]. Although the treatment of LUTS tends to focus on voiding symptoms [1, 2], men typically report storage symptoms (e.g. increased frequency, urgency and nocturia [1, 2]) as the most bothersome [3, 4].

LUTS are commonly associated with benign prostatic hyperplasia (BPH), i.e. LUTS/BPH [1]; together, these conditions have been shown to have a significant impact on men’s health-related quality of life and daily activities [5]. Current European recommendations for treatment of LUTS include the use of lifestyle interventions, and pharmacological therapies when these interventions are inappropriate or unsuccessful (i.e. in men with moderate-to-severe LUTS) [2]. Pharmacological treatment options include α-blockers, antimuscarinics and 5α-reductase inhibitors (5-ARIs), which can either be used as monotherapy or in combination, and recent evidence suggests that the use of pharmacological treatments for LUTS/BPH is increasing in some healthcare systems, particularly as combination therapy [6]. The European recommendations suggest different indications for combination therapy, i.e. α-blocker plus an antimuscarinic if relief of storage symptoms has been insufficient with monotherapy of either drug; and α-blocker plus a 5-ARI in men with a substantially enlarged prostate (those more likely to experience disease progression) [2].

Combination therapies can be administered separately (i.e. as concomitant therapy), or as a fixed-dose combination (FDC) in a single tablet. Several randomised, double-blinded trials conducted in men >40 years of age with LUTS/BPH and overactive bladder (OAB) symptoms have demonstrated improved efficacy in those who received α-blocker plus antimuscarinic combination therapy concomitantly compared with monotherapy [7–10]. With regards to FDCs, a randomised, double-blind, multicentre, phase III study of 1690 men ≥45 years of age with moderate-to-severe LUTS/BPH (NEPTUNE; Clinicaltrials.gov identifier: NCT01018511), demonstrated that a FDC of solifenacin 6 mg plus tamsulosin oral controlled absorption system (TOCAS, 0.4 mg) significantly improved voiding and storage symptoms versus placebo, and storage symptoms versus TOCAS alone [11]. The FDC tablet of solifenacin 6 mg plus TOCAS is approved for the treatment of men with moderate-to-severe LUTS/BPH in Europe [12] and was first authorised for marketing in the Netherlands in May 2013 [13]. A FDC of dutasteride and tamsulosin is approved for use in Europe, but only for men with LUTS and an enlarged prostate [14].

Despite the improvements in symptoms of LUTS/BPH, treatment persistence (i.e. the duration from initiation to discontinuation of therapy [15]) and adherence (i.e. the extent to which a patient acts in accordance with the prescribed interval, and dose of a dosing regimen [15]) are reported to be low in men with LUTS/BPH. An observational study of 8694 men ≥45 years of age with LUTS/BPH conducted in the United Kingdom (UK) showed that 38.5% and 53.0% of men discontinued α-blocker and antimuscarinic therapy, respectively, over a median duration of 2.1 years’ treatment [16]; and a retrospective study of 670 men with LUTS/BPH in Korea found that approximately two-thirds of men discontinued an α-blocker, a 5-ARI, or both treatments in combination within 1 year of starting treatment) [17]. In the latter study, adverse events (AEs) were among the most common reasons for discontinuation and for switching of treatment.

Overall, there are limited published data regarding treatment patterns in men receiving combination therapy for LUTS/BPH in routine clinical practice. The aims of this study were to assess treatment persistence and adherence with α-blocker plus antimuscarinic combination therapy in men with LUTS/BPH, and compare these endpoints with treatment administered either as a FDC or combination therapy given concomitantly.

## Methods

### Study design

This was a retrospective, observational cohort study of men with LUTS/BPH who received prescription(s) for combination therapy with an α-blocker plus an antimuscarinic or a 5-ARI. Anonymised patient longitudinal prescription records and demographic data were extracted from the Netherlands IMS LifeLink™ LRx database, which consists of data from pharmacies and dispensing general practitioners (GPs) in the Netherlands (total sample is representative of around 16.5 million people).

The primary objective of the study was to assess treatment persistence in men who received α-blocker plus antimuscarinic combination therapy when prescribed as a FDC, compared with prescriptions of separate combination drugs (concomitant therapy). Adherence was also assessed in these two comparative groups as a secondary objective. Other secondary objectives included: comparing treatment persistence with an α-blocker plus an antimuscarinic combination therapy in subgroups defined by the antimuscarinic drug prescribed; and determining the impact of patient/clinical characteristics associated with persistence to combination therapy. Exploratory objectives were to determine the proportion of men who switched combination therapy (described below); and to compare treatment persistence and adherence in men prescribed with any FDC versus any concomitant therapy (α-blocker plus antimuscarinic or 5-ARI for both types).

### Study population

Men ≥45 years of age were treated with combination therapy of an α-blocker plus an antimuscarinic or 5-ARI, prescribed either as a FDC or concomitantly (see Additional file 1: Table S1 for eligible drugs). Combination therapy had to be first prescribed between 1 November 2013 and 31 October 2014 (i.e. the selection period) (Additional file 2: Figure S1), prescriptions had to be received on the same day or within a 30-day window, and all men required continuous enrolment 6 months prior to and 12 months after the start of receiving combination therapy. The end date of database interrogation was 31 October 2015. The start or index date was defined as the date of first prescription of combination therapy – if combination drugs were not received on the same day, the index date was the day on which the second drug in combination was first prescribed. Men were excluded if they received only monotherapy for an eligible drug within the selection period or if they were prescribed the same combination therapy on and prior to the index date.

### Endpoints

Treatment persistence (primary endpoint) was defined here as the time from the index date until first discontinuation of at least one of the index combination drugs. The median time to discontinuation, and the proportion of men persistent at 12-months were reported. An index drug was considered discontinued after a period of ≥30 days without prescription renewal; the date of discontinuation was the date of the last prescription of the first discontinued drug in the combination, plus the days of supply of that prescription.

Adherence (secondary endpoint), defined as medical possession ratio (MPR, i.e. the period in which patients have treatment in their possession), was calculated by two methods: the sum of days of supply of the index combination therapy divided by the time to discontinuation (MPR variable) or the sum of days of supply of the index combination therapy divided by 365 days (MPR fixed). The MPR was calculated as mean or median; men were considered as adherent if an MPR of ≥80% was achieved.

Treatment switching (exploratory endpoint), defined as the proportion of men who switched from index combination therapy to another combination therapy (i.e. if at least one drug in the index combination drugs was discontinued and replaced with at least one new drug after the last prescription date, within the 30 days following the discontinuation date).

### Statistical analyses

The main analysis was performed in all men who received an α-blocker plus antimuscarinic, either as FDC or as concomitant therapy. Comparisons of persistence and adherence in this population were performed for FDC α-blocker plus an antimuscarinic compared with concomitant α-blocker plus an antimuscarinic; and α-blocker plus antimuscarinic combination therapy defined by the antimuscarinic agent. An exploratory analysis was performed in men who received any FDC therapy versus any concomitant therapy (α-blocker plus antimuscarinic or 5-ARI in both groups).

Baseline demographics and characteristics were reported descriptively. Time to discontinuation was presented using Kaplan-Meier curves. Treatment persistence and adherence were assessed using multivariate Cox regression models that adjusted for potential confounding factors at index date: age, polypharmacy (number of Anatomical Therapeutic Chemical [ATC3] class drugs [defined by the European Pharmaceutical Market Research Association] excluding those approved for the treatment of LUTS/BPH), and type of prescriber. Adjusted hazard ratios (HRs) with associated confidence intervals (CIs) and *p*-values are reported for comparisons of FDC and concomitant therapy; the FDC was used as the reference. Linear regression models were used for associations of potential confounding factors with MPR.

Several sensitivity analyses of time to discontinuation were performed. One analysis on the definition of time to discontinuation increased the period without prescription renewal from 30 days to 45, 60 and 90 days in the base-case cohort of men (i.e. those who initiated α-blocker and antimuscarinic combination therapy within a 30 day window). Other analyses of time to discontinuation (defined by 30 days without prescription renewal) were performed in men who were treatment-naïve for combination treatment during the pre-index period; men who first received prescriptions for α-blocker and antimuscarinic combination therapy on the same date; and men who first received prescriptions for α-blocker and antimuscarinic combination therapy within an extended 60 day window.

## Results

### Baseline demographics and characteristics

**Fig. 1:**
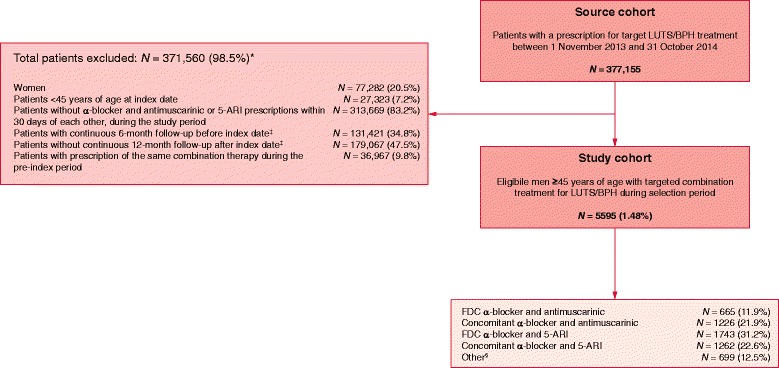
Patient selection flowchart. ^*^Patients excluded by exclusion/inclusion criterion, applied independently from each other; ^‡^A continuous follow-up period was confirmed by the dispensation of any medication 6 months prior to the index date and 12 months following the index date, with no gap in pharmacy records; ^§^Patients with more than two drugs prescribed within 30 days of each other. 5-ARI: 5α-reductase inhibitor; FDC: fixed-dose combination; LUTS/BPH: lower urinary tract symptoms associated with benign prostatic hyperplasia

In total, 377,155 patients were prescribed with a target drug for LUTS/BPH treatment between 1 November 2013 and 31 October 2014. Overall, 371,560 patients were excluded (the most common reason for exclusion was the absence of prescription of an α-blocker and an antimuscarinic or 5-ARI within 30 days of each other during the study period [*N =* 313,669]), leaving a final study population of 5595 eligible men (Fig. 1). Of these, 1891 men received an α-blocker plus an antimuscarinic (665 as FDC and 1226 as concomitant therapy). In those receiving an α-blocker plus an antimuscarinic combination, the most common antimuscarinic was solifenacin (*N =* 1407) and flavoxate the least common (*N =* 23).

Baseline characteristics in the cohort that received an α-blocker plus an antimuscarinic are shown in Table 1. The mean age at index date was 71.95 years and a high proportion of men received α-blocker monotherapy (88.2%) and/or antimuscarinic monotherapy (52.3%) prior to the index date. Baseline characteristics were generally similar when comparing men who received either a FDC or concomitant therapy of an α-blocker plus an antimuscarinic. However, a higher proportion of men prescribed with a FDC compared with the concomitant therapy group had received ≤3 different drug classes for conditions other than LUTS/BPH at baseline (74.7% vs. 53.2%); were prescribed combination therapy at index date by a urologist (68.6% vs. 22.0%); and had received any prior combination therapy (34.6% vs. 20.3%) or 5-ARI monotherapy (13.2 vs. 5.6%). Overall, baseline characteristics were similar in subgroups based on the prescribed antimuscarinic at index date (Additional file 3: Table S2).

**Table 1:** Baseline characteristics^a^ in those receiving combination therapy with an α-blocker plus an antimuscarinic

	Overall population (*N =* 1891)^b^	FDC (*N =* 665)	Concomitant therapy (*N =* 1226)
Age at index date, mean (SD)	71.95 (9.55)	70.46 (9.11)	72.77 (9.68)
Age at index date, *N* (%)			
45–64 years	417 (22.1)	172 (25.9)	245 (20.0)
65–74 years	654 (34.6)	260 (39.1)	394 (32.1)
≥75 years	820 (43.4)	233 (35.0)	587 (47.9)
Polypharmacy,^c^ mean (SD)	3.38 (3.33)	4.35 (3.18)	5.54 (3.40)
Polypharmacy,^c^ *N* (%)			
0	420 (22.2)	223 (33.5)	197 (16.1)
1–3	729 (38.6)	274 (41.2)	455 (37.1)
4–5	303 (16.0)	79 (11.9)	224 (18.3)
6–8	278 (14.7)	68 (10.2)	210 (17.1)
≥9	161 (8.5)	21 (3.2)	140 (11.4)
Prescriber at index date, *N* (%)			
Urologist	726 (38.4)	456 (68.6)	270 (22.0)
GP	931 (49.2)	130 (19.6)	801 (65.3)
Other	234 (12.4)	79 (11.9)	155 (12.6)
Prior treatment, *N* (%)			
Any combination	479 (25.3)	230 (34.6)	249 (20.3)
α-blocker + antimuscarinic	298 (15.8)	121 (18.2)	177 (14.4)
α-blocker	1668 (88.2)	549 (82.6)	1119 (91.3)
Antimuscarinic	989 (52.3)	237 (35.6)	752 (61.3)
5-ARI	157 (8.3)	88 (13.2)	69 (5.6)
Concomitant therapy, *N* (%)			
Both drugs initiated on the same date	–	–	341 (27.8)
Both drugs initiated within 30 days	–	–	885 (72.2)

*5-ARI* 5α-reductase inhibitor, *FDC* fixed-dose combination, *GP* general practitioner, *SD* standard deviation

^a^At index date

^b^The overall population comprised men receiving FDC or concomitant therapy of an α-blocker and an antimuscarinic

^c^Number of drugs (classified by Anatomical Therapeutic Chemical code) prescribed, excluding those approved for the treatment of LUTS/BPH.

### α-blocker plus antimuscarinic: FDC versus concomitant therapy

#### Overall time to discontinuation

**Fig. 2.**
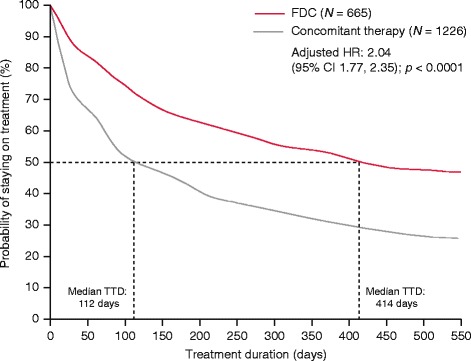
Median time to discontinuation for FDC versus concomitant therapy α-blocker plus antimuscarinic. CI: confidence intervals; FDC: fixed-dose combination; HR: hazard ratio; TTD: time to discontinuation

Median time to discontinuation was significantly longer with α-blocker plus antimuscarinic FDC versus concomitant therapy (414 vs. 112 days; adjusted HR 2.04, 95% CI 1.77, 2.35; *p <* 0.0001) (Fig. 2) and the proportion of men persistent at 12 months was higher with FDC compared with concomitant therapy (51.3% vs. 29.9%).

#### Impact of patient/clinical characteristics on time to discontinuation

**Fig. 3:**
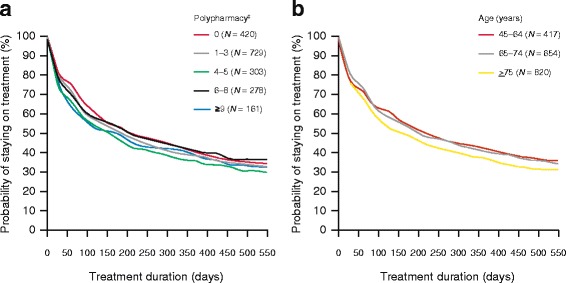
Median time to discontinuation according to polypharmacy^‡^ (**a**) and age (years) at index date (**b**) for all men who received an α-blocker plus antimuscarinic combination therapy (FDC or concomitant therapy). ^‡^Number of drugs (classified by Anatomical Therapeutic Chemical code) prescribed, excluding those approved for the treatment of LUTS/BPH. FDC: fixed-dose combination; LUTS/BPH: lower urinary tract symptoms associated with benign prostatic hyperplasia

Median time to discontinuation and persistence at 12 months were greatest in men aged 45–64 years (217 days and 40.3%) compared with those aged 65–74 years (189 days and 38.5%) and ≥75 years (150 days and 35.1%), although the differences were not statistically significant (Fig. 3). When the results were stratified by the number of drugs received at index date, no significant patterns were observed for median time to discontinuation (range, 153–207 days) and persistence at 12 months (range, 34.0%–40.3%) (Fig. 3; Additional file 4: Table S3). Similarly, no significant differences were observed for median time to discontinuation and persistence at 12 months in men prescribed by a urologist (234 days and 41.5%) compared with those prescribed by a GP (148 days and 35.1%; adjusted HR 0.89, 95% CI 0.78, 1.02; *p =* 0.095) or those prescribed by other healthcare providers (181 days and 34.2%; adjusted HR 1.00, 95% CI 0.83, 1.20; *p =* 0.976).

#### Adherence

Mean MPR and the proportion of men adherent at 12 months were similar for men who received FDC and concomitant therapy (Table 2). Similar adherence data in the FDC and concomitant therapy groups were also evident in the subgroup of men who were persistent at 12 months.

**Table 2:** Adherence in men receiving FDC and concomitant therapy with an α-blocker plus an antimuscarinic

	FDC α-blocker and antimuscarinic	Concomitant therapy	
		α-blocker	antimuscarinic
MPR-fixed			
*N*	566	726	726
Mean (SD)	0.91 (0.52)	0.95 (0.37)	0.89 (0.31)
Adherent,^a^ *n* (%)	453 (80.0)	623 (85.8)	546 (75.2)
MPR-variable			
*N*	566	726	726
Mean (SD)	0.67 (0.31)	0.69 (0.34)	0.64 (0.32)
Adherent,^a^ *n* (%)	275 (48.6)	358 (49.3)	319 (43.9)
MPR-fixed (persistent men only)			
*N*	313	380	380
Mean (SD)	0.83 (0.21)	0.93 (0.22)	0.85 (0.22)
Adherent,^a^ *n* (%)	235 (75.1)	324 (85.3)	282 (74.2)

*FDC* fixed-dose combination, *MPR* medical possession ratio, *SD* standard deviation

^a^MPR of ≥80%

#### Sensitivity analyses

Compared with the base-case analysis of 30 days, adjusting the time used to define discontinuation of combination therapy to 45, 60 and 90 days had minimal impact on the results for concomitant therapy but increased median time to discontinuation for the FDC (Additional file 5: Table S4). At each of these timepoints, median time to discontinuation and persistence at 12 months were significantly greater in men who received FDC compared with concomitant therapy (*p <* 0.001 for all assessments). In treatment-naïve men, median time to discontinuation (384 vs. 113 days) and persistence at 12 months (49.6% vs. 30.9%) were significantly greater in those who received FDC versus concomitant therapy (adjusted HR 1.83, 95% CI 1.60, 2.09; *p <* 0.0001) (Additional file 6: Figure S2a). Median time to discontinuation (414 vs. 198 days) and persistence at 12 months (51.3% vs. 38.7%) were also significantly greater in the FDC group compared with men prescribed concomitant combination treatment on the same day (adjusted HR 1.46, 95% CI 1.23, 1.72; *p <* 0.0001) (Additional file 6: Figure S2b). Similarly, in men who received prescriptions for α-blocker and antimuscarinic combination therapy within a 60 day window, median time to discontinuation (424 vs. 90 days) and persistence at 12 months (52.2% vs. 25.7%) were significantly greater in those who received FDC versus concomitant therapy (adjusted HR 2.17; 95% CI 1.90, 2.48; *p <* 0.0001) (Additional file 6: Figure S2c).

#### Switching

Among the 1183 men who were non-persistent at 12 months, a similar proportion of men who received FDC therapy of an α-blocker plus antimuscarinic switched treatment at 12 months, compared with those receiving concomitant therapy (5.9% vs. 6.5%, respectively) (Table 3), with the majority of men discontinuing treatment and not receiving a prescription for a new combination within 30 days. Due to the low number of men who switched treatment, no clear patterns were observed for the type of treatment men subsequently switched to.

**Table 3:** Change of treatment in men initially prescribed an α-blocker plus an antimuscarinic combination treatment and non-persistent at 12 months

	FDC	Concomitant therapy
Change of treatment in men non-persistent at 12 months, *N* (%)	*N =* 324	*N =* 859
Switch^a^	19 (5.9)	56 (6.5)
No switch/discontinuation^b^	305 (94.1)	803 (93.5)
Switched to, *N* (%)	*N =* 19	*N =* 56
Combination with new α-blocker	1 (5.3)	5 (8.9)
Combination with new antimuscarinic	6 (31.6)	25 (44.6)
Concomitant therapy with the same drugs	9 (47.4)	3 (5.4)
Concomitant therapy with a new α-blocker and antimuscarinic	3 (15.8)	0
FDC	0	23 (41.1)
No switch/discontinuation, *N* (%)	*N =* 305	*N =* 803
α-blocker therapy only	58 (19.0)	246 (30.6)
Antimuscarinic therapy only	19 (6.2)	107 (13.3)
No therapy changes	228 (74.8)	450 (56.0)

*FDC* fixed-dose combination

^a^Alternative combination therapy prescribed within 30 days following discontinuation of index combination therapy

^b^No alternative combination therapy prescribed within 30 days following discontinuation of index combination therapy

### α-blocker plus antimuscarinic: antimuscarinic drug subgroups

#### Overall time to discontinuation

**Fig. 4:**
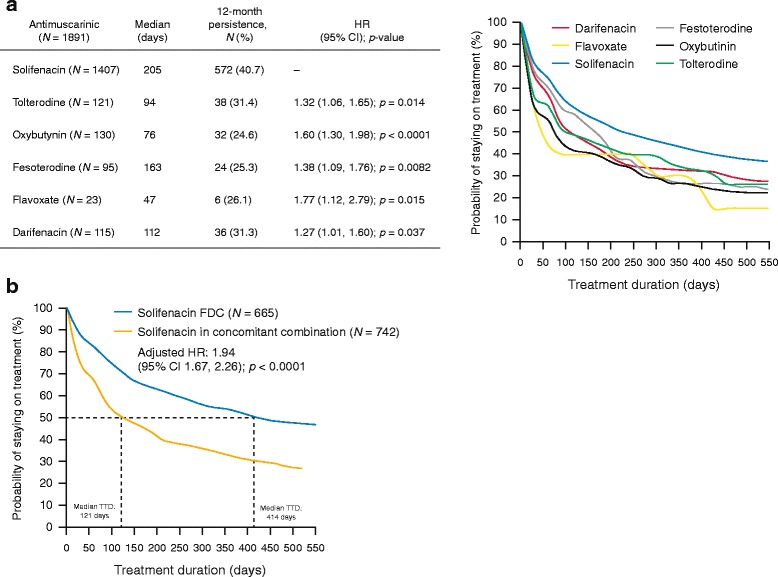
Median time to discontinuation according to the antimuscarinic used in combination (α-blocker and antimuscarinic, either as FDC or concomitant therapy) (**a**) and for α-blocker plus solifenacin (FDC vs. concomitant) combination therapy (**b**). CI: confidence intervals; FDC: fixed-dose combination; HR: hazard ratio; TTD: time to discontinuation

Median time to discontinuation was significantly longer with FDC or concomitant combination therapies containing an α-blocker plus solifenacin (214 days), compared with other α-blocker plus antimuscarinic combination therapies (range, 47–164 days; adjusted HR range 1.27–1.77, *p* = 0.037) (Fig. 4a). Similarly, the proportion of men persistent at 12 months was higher with FDC or concomitant combination therapies containing an α-blocker plus solifenacin (40.7%) compared with other α-blocker plus antimuscarinic combinations (range, 24.6%–31.4%). In the subgroup of men who received an α-blocker plus solifenacin, median time to discontinuation (414 vs. 121 days; adjusted HR 1.94, 95% CI 1.67, 2.26; *p <* 0.0001) and persistence at 12 months (51.3% vs. 31.1%) were significantly greater in those treated with FDC compared with concomitant therapy (Fig. 4b).

#### Switching

Among the 75 men who switched treatment, a similar proportion of men switched from solifenacin compared with other antimuscarinics (6.9% vs. range, 2.8–6.1%).

### α-blocker plus antimuscarinic or 5-ARI: FDC versus concomitant therapy

#### Overall time to discontinuation

A similar number of men received any FDC (*N =* 2408) or any concomitant therapy (*N =* 2488). The median time to discontinuation was significantly longer in men who received any FDC versus any concomitant combination treatment (not reached vs. 193 days; adjusted HR 2.28, 95% CI 2.10, 2.48; *p <* 0.0001) (Additional file 7: Figure S3); persistence at 12 months was also greater (62.1% vs. 38.1%) in the FDC group.

## Discussion

This retrospective study assessed treatment persistence and adherence in over 5000 men with LUTS/BPH and was the first comparison in this population of men receiving treatment with an α-blocker and an antimuscarinic, either as a FDC or as concomitant therapy. Overall, treatment persistence was significantly greater in men who received an α-blocker plus an antimuscarinic as a FDC tablet compared with an α-blocker plus an antimuscarinic given concomitantly. Treatment adherence (assessed by MPR-fixed and -variable) was similar in men who received FDC α-blocker plus antimuscarinic compared with concomitant therapy.

The main study results of improved treatment persistence with FDC α-blocker plus antimuscarinic compared with concomitant therapy were also observed in the sensitivity analyses, which tested adjusting the gaps used to define time to discontinuation. Similar findings were also observed in the subpopulations of treatment-naïve men, those prescribed concomitant combination treatment on the same day, and men who initiated α-blocker and antimuscarinic combination therapy within a 60 day window. Overall, these findings were similar to the base-case analysis and the HRs reported were stable, suggesting that the study results are robust.

The foundation of superior treatment persistence with FDC α-blocker plus antimuscarinic observed in this study is likely to be multifactorial. The results may be partly attributable to the convenience of taking a single tablet (i.e. the FDC) compared with taking two tablets concomitantly. Indeed, patients receiving anti-hypertensive FDC therapy compared with concomitant therapy have reported significantly increased persistence and adherence in a large, retrospective cohort analysis [18]. In contrast, results from a recent retrospective, population-based cohort study, conducted using prescription records and hospital discharge codes from ~1.5 million men with LUTS/BPH in Italy showed that men were significantly less adherent to, and more likely to discontinue treatment with combination therapy of an α-blocker plus a 5-ARI, compared with monotherapy with either treatment, over 5-years of follow-up [19]. However, there are several notable differences to the current study: Cindolo et al. assessed different drug classes (α-blockers and 5-ARIs), defined discontinuation as no prescriptions for at least two consecutive months, and comparisons were made between combination therapy and the two monotherapies, rather than between FDC and concomitant therapies. In the current study, approximately 40% of the men eligible for inclusion were receiving six or more other drugs types at index date and therefore it is difficult to make conclusions about the impact of convenience from a single tablet on the overall results.

Although the efficacy/tolerability of a FDC has not been directly compared with concomitant combination treatment for LUTS/BPH, studies in other indications have reported improved efficacy and tolerability with FDCs compared with concomitant therapies [20, 21]. The efficacy of FDC therapy in male LUTS has been shown in several studies, for instance, significant improvements in Total Urgency and Frequency Score (TUFS) was observed for FDC solifenacin 6 mg plus TOCAS versus TOCAS alone (*p =* 0.025) in the NEPTUNE study of 1334 men with storage and voiding LUTS/BPH [11]. In this study, no improvements in efficacy were observed when comparing the FDC solifenacin 6 mg plus TOCAS versus FDC solifenacin 9 mg plus TOCAS. However, our study did not account for different dose strengths or formulations of antimuscarinics used in combination, therefore similar conclusions cannot be drawn with regards to persistence or adherence. An open-label extension of the NEPTUNE trial (NEPTUNE II) demonstrated that FDC solifenacin 6 mg plus TOCAS was well tolerated (most AEs were mild or moderate) and reductions in International Prostate Symptom Score (IPSS) and TUFS were maintained for up to 52 weeks [22]. In addition, the results from a randomised, open-label, 24-month parallel-group study of 742 men with moderately symptomatic BPH showed significant improvements in IPSS (−5.4 vs. –3.6 [*p <* 0.001]) in men who received FDC dutasteride 0.5 mg plus tamsulosin 0.4 mg versus tamsulosin 0.4 mg (initiated in men whose symptoms did not improve with watchful waiting) [23].

FDC solifenacin 6 mg plus TOCAS 0.4 mg is the only FDC α-blocker plus antimuscarinic approved for the treatment of men with LUTS/BPH [12, 14] and the inclusion of solifenacin within this FDC examined in our study may have contributed to the main findings. Previously reported data from a large, retrospective observational cohort study of 8694 men with LUTS/BPH in the UK showed that fewer patients discontinued (43.0% vs. [mean] 53.0%) or switched treatment (15.3% vs. [mean] 22.0%) from solifenacin compared with most other antimuscarinics, and persisted on-treatment for longer (median duration, 90 days vs. [range] 30–116 days) [16]. These findings are supported by further real-world data in patients with OAB, suggesting that solifenacin provides greater treatment persistence compared with other antimuscarinics (mean persistence 187 vs. 77–157 days; persistence at 12 months, 35% vs. 14%–28%) [24]. The reasons for improved persistence with solifenacin relative to other antimuscarinics are likely attributable to the favourable efficacy and tolerability profile for solifenacin. A long-term open label study of solifenacin for up to 1 year reported that 81% of patients completed 40 weeks of treatment and only 4.7% of patients discontinued treatment due to AEs in patients with OAB [25]. A network meta-analysis of randomised controlled trials conducted in adult patients with OAB showed that solifenacin 5 mg/day provides similar or better efficacy, and a lower or similar risk of dry mouth compared with other common oral antimuscarinics [26]. Patients’ perceptions of symptom control/bother may also be a factor in a decision to persist with or discontinue treatment. Patients receiving solifenacin for the treatment of OAB have reported significant improvements in health-related quality of life and perceived bother compared with active comparator treatment or placebo [27, 28]. Indeed, unmet treatment expectations and/or tolerability are the primary reasons for treatment discontinuation in up to 90% of non-persistent patients [29]. In our study, although time to discontinuation and persistence were greater in patients receiving FDC or concomitant combinations containing solifenacin versus other antimuscarinics, the influence of solifenacin on switching could not be assessed due to low numbers of men (*N =* 75) who met the switching criteria (i.e. replacing a discontinued index drug with at least one new drug within 30 days of the discontinuation date).

Baseline characteristics were well balanced at the index date and few significant differences were observed when the results for persistence were stratified by age, polypharmacy and prescriber. These data suggest that further study is needed to identify the key drivers of persistence in men receiving combination therapy for LUTS/BPH. In particular, it is hard to draw conclusions regarding the true effects of polypharmacy from the results, as men may have been receiving other treatments for a number of different conditions, and the definition of treatment-naïve men was only applied for 6 months prior to the study commencing (as such, the number of previous therapies for LUTS/BPH, including prior receipt of combination therapy, and the time since diagnosis could not be determined). There was a trend (not statistically significant) for greater persistence in men who received prescriptions from urologists compared with GPs. This finding is supported by recent evidence from a retrospective cohort study of 252 men with OAB, which reported that persistence on treatment was higher among men receiving subspecialist supervision, compared with those receiving treatment in internal medicine or general urology departments [30].

Strengths of this study include a large sample of approximately 5000 men with LUTS/BPH and results based on real-world data, using prescription records from a representative sample of pharmacies and dispensing GPs (corresponding to approximately 75% of retail dispensing in the Netherlands). Although real-word data were used, no in-depth clinical information was available regarding diagnosis or reasons for stopping treatment and this was a limitation. Other limitations of the database were that no information was reported on whether men received repeat prescriptions in other pharmacies outside the panel, moved to another address or died (although this was partly addressed by defining the post-index period based on the last available information on other medications). If a patient filed prescriptions at different pharmacies (i.e. one which was not included in the panel), this resulted in missing medication history and misclassification of patients. However, evidence suggests that >90% of patients in the Netherlands are usually loyal to one pharmacy [31]. Also, no information was available about whether drugs were taken correctly, according to the treatment regimen; persistence and adherence were calculated based on the recorded duration of treatment and it was assumed that when a patient was prescribed a medication then it was indeed picked up and used by the patient (however, this limitation is not restricted to retrospective analyses). Therefore, persistence and adherence rates may have been overestimated due to this assumption, although this would have been equivalent in all subcohorts and should not have influenced the comparative results. Although a reasonable number of patients (>100) were prescribed with tolterodine, oxybutynin and darifenacin, solifenacin was the most commonly prescribed antimuscarinic in the primary cohort of our study, and this may have influenced the results of the antimuscarinic subgroup analyses. Observational studies of treatment persistence/adherence with antimuscarinic therapy conducted in the UK and Canada also reported that solifenacin was the most commonly prescribed medication for OAB [24, 32, 33]. However, the pattern of prescriptions across the antimuscarinics was more balanced in these studies, suggesting that the proportion of patients prescribed with solifenacin in the current study (74.4%) may be specific to the Netherlands. Regarding α-blockers, tamsulosin is reported to be the most commonly used for LUTS/BPH [16], but it should also be noted that the impact of individual α-blockers used in combination on persistence, adherence or switching were not assessed in the same way as antimuscarinics in this study; this could perhaps be evaluated in further studies.

Future qualitative studies could also explore the rationale for treatment persistence/switching in LUTS/BPH and the potential benefits which are derived from improved persistence, particularly with regards to efficacy and tolerability, and also healthcare resource use and cost-effectiveness. In such analyses, it should be taken into consideration that patients’ medication-taking behaviour (i.e. persistence or adherence) can be attributed to a number of factors, including side effects experienced on-treatment [34, 35], the patient’s beliefs, values [35] and perception of the severity of their condition [34], and other behavioural or societal factors [34].

## Conclusion

This study suggests that men with LUTS/BPH receiving α-blocker plus antimuscarinic combination therapy as a FDC remain on treatment significantly longer and have superior rates of persistence and adherence compared with men receiving concomitant therapy of an α-blocker plus antimuscarinic. These findings may be useful for prescribers, as improved persistence on-treatment may translate to improved outcomes for men with LUTS/BPH. Further study is warranted to assess the key drivers of persistence in men receiving combination therapy for LUTS/BPH, and also to establish the effects of such therapies on efficacy and tolerability in this patient population.

## Additional files

Additional file 1: Table S1. Drugs available for selection using European Pharmaceutical Market Research Association (EphMRA) ATC drug codes [36]. (DOCX 13 kb)

Additional file 2: Figure S1. Study design. (PDF 1 mb)

Additional file 3: Table S2. Baseline characteristics* in all men who received combination therapy with an α-blocker plus an antimuscarinic, according to the antimuscarinic drug prescribed (*N =* 1891) (DOCX 14 kb)

Additional file 4: Table S3. Persistence in all men who received an α-blocker plus an antimuscarinic (*N =* 1891): multivariate analysis adjusting baseline characteristics* (DOCX 13 kb)

Additional file 5: Table S4. Persistence in all men who received an α-blocker blocker plus an antimuscarinic (*N* = 1891): sensitivity analysis adjusting the gap lengths used to define discontinuation. (DOCX 13 kb)

Additional file 6: Figure S2. Median time to discontinuation: sensitivity analyses of treatment-naïve men (no prior combination therapy)* (a); index combination first prescribed on the same date* (b); and men who received an α-blocker and an antimuscarinic as combination therapy within 60 days 635 ^‡^ (c). (PDF 962 kb)

Additional file 7: Figure S3. Median time to discontinuation in any FDC* compared with any concomitant therapy*. (PDF 945 kb)

## Abbreviations

5-ARIs: 5α-reductase inhibitors
AEs: Adverse events
BPH: Benign prostatic hyperplasia
CI: Confidence interval
FDC: Fixed-dose combination
GP: General practitioner
HR: Hazard ratio
IPSS: International Prostate Symptom Score
LUTS: Lower urinary tract symptoms
MPR: Medical possession ratio
OAB: Overactive bladder
TOCAS: Tamsulosin oral controlled absorption system
TUFS: Total Urgency and Frequency Score
