# Supplementary material for: A retrospective study of treatment persistence and adherence to α-blocker plus antimuscarinic combination therapies, in men with LUTS/BPH in the Netherlands
Source: BMC Urol. 2017 May 22;17:36. doi: 10.1186/s12894-017-0226-2 (PMC5440896; doi:10.1186/s12894-017-0226-2)
Supplement: Supplementary file 1 — Drugs available for selection using European Pharmaceutical Market Research Association (EphMRA) ATC drug codes [36]. (DOCX 13 kb) [file 12894_2017_226_MOESM1_ESM.docx]

**Table S1.** Drugs available for selection using European Pharmaceutical Market Research Association (EphMRA) ATC drug codes [36]

| Drug | ATC code |
| --- | --- |
| α-blocker (concomitantly in combination with antimuscarinic or 5-ARI) |  |
| Prazosin | C02CA01 |
| Indoramin | C02CA02 |
| Trimazosin | C02CA03 |
| Doxazosin | C02CA04 |
| Urapidil | C02CA06 |
| Alfuzosin | G04CA01 |
| Tamsulosin | G04CA02 |
| Terazosin | G04CA03 |
| Silodosin | G04CA04 |
| Antimuscarinic (concomitantly in combination with α-blocker) |  |
| Emepronium | G04BD01 |
| Flavoxate | G04BD02 |
| Meladrazine | G04BD03 |
| Oxybutynin | G04BD04 |
| Terodiline | G04BD05 |
| Propiverine | G04BD06 |
| Tolterodine | G04BD07 |
| Solifenacin | G04BD08 |
| Trospium | G04BD09 |
| Darifenacin | G04BD10 |
| Fesoterodine | G04BD11 |
| 5-ARI (concomitantly in combination with α-blocker) |  |
| Finasteride | G04CB01 |
| Dutasteride | G04CB02 |
| FDC |  |
| Alfuzosin and finasteride | G04CA51 |
| Tamsulosin and dutasteride | G04CA52 |
| Tamsulosin and solifenacin | G04CA53 |

5-ARI: 5α-reductase inhibitor; ATC: Anatomical Therapeutic Chemical classification system; FDC: fixed-dose combination

Reference

36. World Health Organization: ATC/DDD Index 2016. Available at: <http://www.whocc.no/atc_ddd_index/> (Access date: 8 Nov 2016).
