# Supplementary material for: A retrospective study of treatment persistence and adherence to α-blocker plus antimuscarinic combination therapies, in men with LUTS/BPH in the Netherlands
Source: BMC Urol. 2017 May 22;17:36. doi: 10.1186/s12894-017-0226-2 (PMC5440896; doi:10.1186/s12894-017-0226-2)
Supplement: Supplementary file 2 — Study design. (PDF 1 mb) [file 12894_2017_226_MOESM2_ESM.pdf]

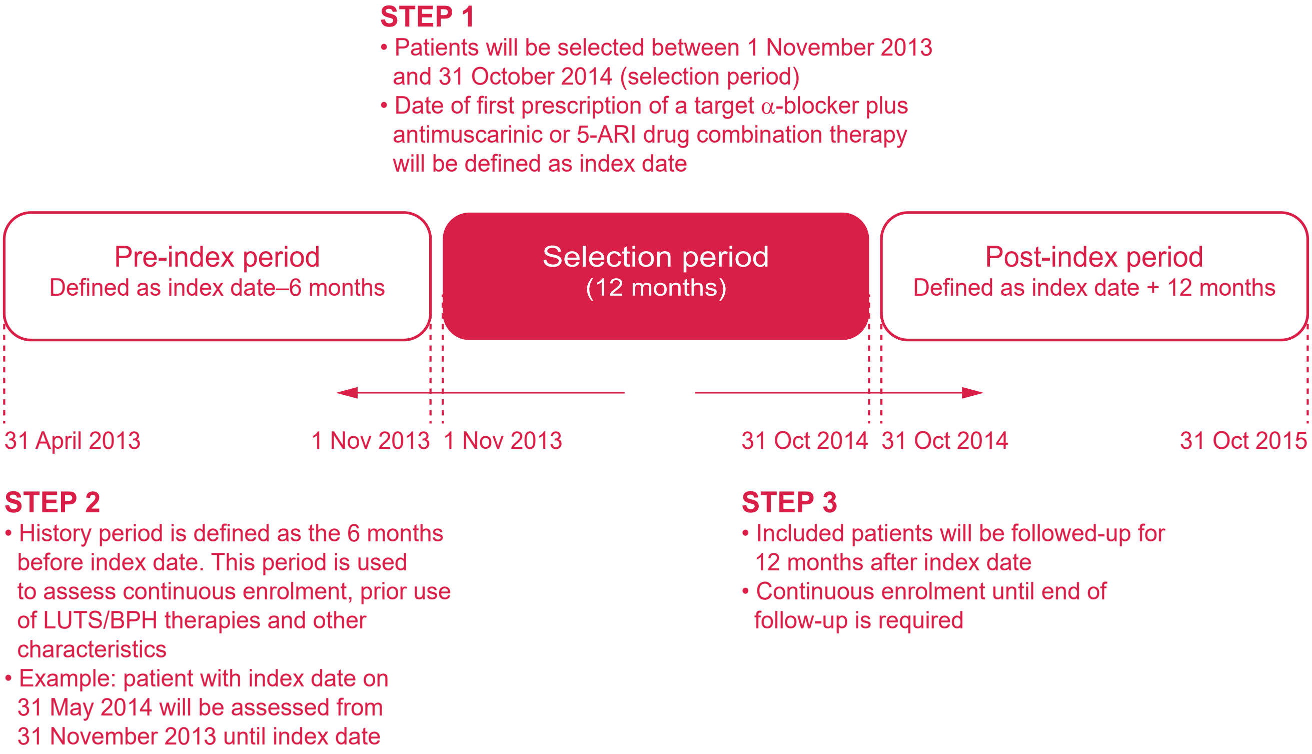

Figure S1. Study design. 5-ARI: 5α-reductase inhibitor; FDC: fixed-dose combination; LUTS/BPH: lower urinary tract symptoms associated with benign prostatic hyperplasia
