# Supplementary material for: A retrospective study of treatment persistence and adherence to α-blocker plus antimuscarinic combination therapies, in men with LUTS/BPH in the Netherlands
Source: BMC Urol. 2017 May 22;17:36. doi: 10.1186/s12894-017-0226-2 (PMC5440896; doi:10.1186/s12894-017-0226-2)
Supplement: Supplementary file 3 — Baseline characteristics* in all men who received combination therapy with an α-blocker plus an antimuscarinic, according to the antimuscarinic drug prescribed (N = 1891) (DOCX 14 kb) [file 12894_2017_226_MOESM3_ESM.docx]

**Table S2.** Baseline characteristics^a^ in all men who received combination therapy with an α-blocker plus an antimuscarinic, according to the antimuscarinic drug prescribed (*N =* 1891)

|  | Solifenacin (*N =* 1407) | Tolterodine (*N =* 121) | Oxybutynin (*N =* 130) | Fesoterodine (*N =* 95) | Flavoxate  (*N =* 23) | Darifenacin (*N =* 115) |
| --- | --- | --- | --- | --- | --- | --- |
| Age, mean years (SD) | 71.57 (9.43) | 73.29 (9.64) | 73.42 (10.23) | 70.96 (9.62) | 73.74 (10.08) | 74.08 (9.45) |
| Age group, *N* (%) | | | | | | |
| 45–64 years | 322 (22.9) | 19 (15.7) | 24 (18.5) | 27 (28.4) | 5 (21.7) | 20 (17.4) |
| 65–74 years | 495 (35.2) | 43 (35.5) | 43 (33.1) | 32 (33.7) | 6 (26.1) | 45 (39.1) |
| ≥75 years | 590 (41.9) | 59 (48.8) | 63 (48.5) | 36 (37.9) | 12 (52.2) | 60 (52.2) |
| Polypharmacy, mean (SD)^‡^ | 3.10 (3.18) | 3.67 (3.26) | 4.77 (4.08) | 3.33 (2.95) | 4.70 (4.55) | 4.70 (3.63) |
| Polypharmacy, *N* (%)^b^ | | | | | | |
| 0 | 353 (25.1) | 20 (16.5) | 17 (13.1) | 17 (17.9) | 1 (4.3) | 5 (4.3) |
| 1–3 | 552 (39.2) | 46 (38.0) | 40 (30.8) | 41 (43.2) | 12 (52.2) | 38 (33.0) |
| 4–5 | 211 (15.0) | 23 (19.0) | 25 (19.2) | 18 (18.9) | 4 (17.4) | 22 (19.1) |
| 6–8 | 193 (13.7) | 20 (16.5) | 25 (19.2) | 14 (14.7) | 3 (13.0) | 23 (20.0) |
| >9 | 98 (7.0) | 12 (9.9) | 23 (17.7) | 5 (5.3) | 3 (13.0) | 20 (17.4) |
| Prescriber, *N* (%) | | | | | | |
| Urologist | 629 (44.7) | 14 (11.6) | 17 (13.1) | 29 (30.5) | 1 (4.3) | 36 (31.3) |
| GP | 608 (43.2) | 89 (73.6) | 90 (69.2) | 60 (63.2) | 19 (82.6) | 65 (56.5) |
| Other | 170 (12.1) | 18 (14.9) | 23 (17.7) | 6 (6.3) | 3 (13.0) | 14 (12.2) |
| Prior combination therapy, *N* (%) | 339 (24.1) | 17 (14.0) | 33 (25.4) | 42 (44.2) | 6 (26.1) | 42 (36.5) |
| α-blocker | 1234 (87.7) | 104 (86.0) | 117 (90.0) | 88 (92.6) | 22 (95.7) | 103 (89.6) |
| Antimuscarinic | 658 (46.8) | 75 (62.0) | 87 (66.9) | 76 (80.0) | 14 (60.9) | 79 (68.7) |
| 5-ARI | 131 (9.3) | 4 (3.3) | 9 (6.9) | 6 (6.3) | 0 | 7 (6.1) |

5-ARI: 5α-reductase inhibitor; ATC: Anatomical Therapeutic Chemical; GP: general practitioner; SD: standard deviation

^a^At index date

^b^Number of drugs (classified by ATC code) prescribed, excluding those approved for the treatment of LUTS/BPH
