# Supplementary material for: A retrospective study of treatment persistence and adherence to α-blocker plus antimuscarinic combination therapies, in men with LUTS/BPH in the Netherlands
Source: BMC Urol. 2017 May 22;17:36. doi: 10.1186/s12894-017-0226-2 (PMC5440896; doi:10.1186/s12894-017-0226-2)
Supplement: Supplementary file 4 — Persistence in all men who received an α-blocker plus an antimuscarinic (N = 1891): multivariate analysis adjusting baseline characteristics* (DOCX 13 kb) [file 12894_2017_226_MOESM4_ESM.docx]

**Table S3.** Persistence in all men who received an α-blocker plus an antimuscarinic (*N =* 1891): multivariate analysis adjusting baseline characteristics^a^

|  | Age group, years | | |  | Polypharmacy^b^ | | | | |  | Prescriber | | |
| --- | --- | --- | --- | --- | --- | --- | --- | --- | --- | --- | --- | --- | --- |
|  | 45–65  (*N =* 417) | 65–74  (*N =* 654) | ≥75  (*N =* 820) |  | 0  (*N =* 420) | 1–3  (*N =* 729) | 4–5  (*N =* 303) | 6–8  (*N =* 278) | ≥9  (*N =* 161) |  | Urologist (*N =* 726) | GP  (*N =* 931) | Other  (*N =* 234) |
| Time to discontinuation | |  |  |  |  |  |  |  |  |  |  |  |  |
| Median (days) | 217 | 189 | 150 |  | 207 | 182 | 153 | 199 | 154 |  | 234 | 148 | 181 |
| IQR | 50, – | 58, – | 32.5, – |  | 60, – | 52, – | 30, – | 45, – | 30, – |  | 63, – | 30, – | 45, – |
| HR (95% CI) | – | 1.02 (0.88, 1.19) | 1.11 (0.96, 1.29) |  | – | 0.95 (0.82, 1.10) | 0.96 (0.80, 1.16) | 0.81 (0.67, 0.99)^c^ | 0.87 (0.69, 1.10) |  | – | 0.89 (0.78, 1.02) | 1.00 (0.83, 1.20) |
| 12-month persistence, *N* (%) | 168 (40.3) | 252 (38.5) | 288 (35.1) |  | 160 (38.1) | 273 (37.4) | 103 (34.0) | 112 (40.3) | 60 (37.3) |  | 301 (41.5) | 327 (35.1) | 80 (34.2) |

ATC: Anatomical Therapeutic Chemical; BPH: benign prostatic hyperplasia; CI: confidence intervals; GP: general practitioner; HR: hazard ratio; IQR: interquartile range; LUTS: lower urinary tract symptoms

^a^At index date

^b^Number of drugs (classified by ATC code) prescribed, excluding those approved for the treatment of LUTS/BPH

^c^*p* < 0.05
