# Supplementary material for: A retrospective study of treatment persistence and adherence to α-blocker plus antimuscarinic combination therapies, in men with LUTS/BPH in the Netherlands
Source: BMC Urol. 2017 May 22;17:36. doi: 10.1186/s12894-017-0226-2 (PMC5440896; doi:10.1186/s12894-017-0226-2)
Supplement: Supplementary file 5 — Persistence in all men who received an α-blocker blocker plus an antimuscarinic (N = 1891): sensitivity analysis adjusting the gap lengths used to define discontinuation. (DOCX 13 kb) [file 12894_2017_226_MOESM5_ESM.docx]

**Table S4.** Persistence in all men who received an α-blocker plus an antimuscarinic (*N =* 1891): sensitivity analysis adjusting the gap lengths used to define discontinuation

|  | 30 days gap (base case) | |  | 45 days gap | |  | 60 days gap | |  | 90 days gap | |
| --- | --- | --- | --- | --- | --- | --- | --- | --- | --- | --- | --- |
|  | FDC  (*N =* 665) | Concomitant therapy  (*N =* 1226) |  | FDC  (*N =* 665) | Concomitant therapy  (*N =* 1226) |  | FDC  (*N =* 665) | Concomitant therapy  (*N =* 1226) |  | FDC  (*N =* 665) | Concomitant therapy  (*N =* 1226) |
| Time to discontinuation |  |  |  |  |  |  |  |  |  |  |  |
| Median (days) | 414 | 112 |  | 500 | 113 |  | 602 | 114 |  | NR | 115 |
| IQR | 100, – | 30, 553 |  | 104, – | 30, 628 |  | 105, – | 30, – |  | 108, – | 30, – |
| HR (95% CI) | 2.04  (1.77, 2.35)^a^ | |  | 2.13  (1.85, 2.46) ^a^ | |  | 2.15  (1.86, 2.49) ^a^ | |  | 2.24  (1.93, 2.60) ^a^ | |
| 12-month persistence, *N* (%) | 341  (51.3) | 367  (29.9) |  | 368  (55.3) | 384  (31.3) |  | 376  (56.5) | 396  (32.3) |  | 391  (58.8) | 402  (32.8) |

CI: confidence intervals; FDC: fixed-dose combination; HR: hazard ratio; IQR: interquartile range; NR: not reached

^a^*p* < 0.001
