# Supplementary material for: A retrospective study of treatment persistence and adherence to α-blocker plus antimuscarinic combination therapies, in men with LUTS/BPH in the Netherlands
Source: BMC Urol. 2017 May 22;17:36. doi: 10.1186/s12894-017-0226-2 (PMC5440896; doi:10.1186/s12894-017-0226-2)
Supplement: Supplementary file 6 — Median time to discontinuation: sensitivity analyses of treatment-naïve men (no prior combination therapy)* (a); index combination first prescribed on the same date* (b); and men who received an α-blocker and an antimuscarinic as combination therapy within 60 days 635 ‡ (c). (PDF 962 kb) [file 12894_2017_226_MOESM6_ESM.pdf]

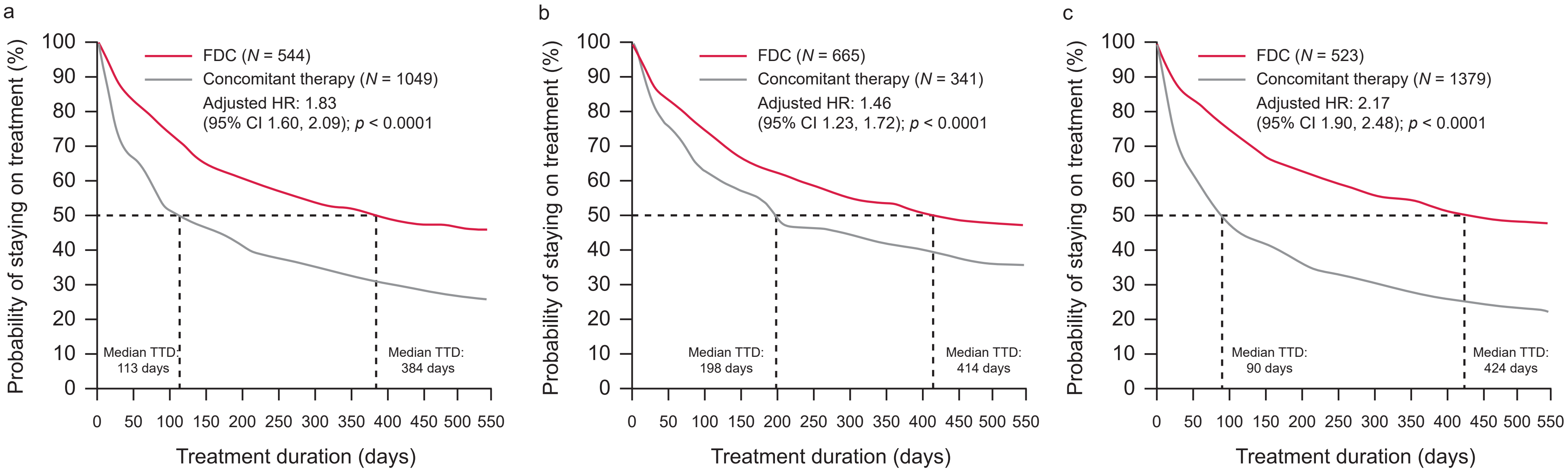


**Figure S2.** Median time to discontinuation: sensitivity analyses of treatment-naïve men (no prior combination therapy)* (a); index combination first prescribed on the same date* (b); and men who received an α-blocker an an antimuscarinic as combination therapy within 60 days‡ (c). *Men who received α-blocker plus antimuscarinic; ‡Men who initiated α-blocker and antimuscarinic combination therapy within a 60 day window. 5-ARI: 5α-reductase inhibitor; CI: confidence intervals; FDC: fixed-dose combination; HR: hazard ratio; TTD: time to discontinuation
