# Supplementary material for: A retrospective study of treatment persistence and adherence to α-blocker plus antimuscarinic combination therapies, in men with LUTS/BPH in the Netherlands
Source: BMC Urol. 2017 May 22;17:36. doi: 10.1186/s12894-017-0226-2 (PMC5440896; doi:10.1186/s12894-017-0226-2)
Supplement: Supplementary file 7 — Median time to discontinuation in any FDC* compared with any concomitant therapy*. (PDF 945 kb) [file 12894_2017_226_MOESM7_ESM.pdf]

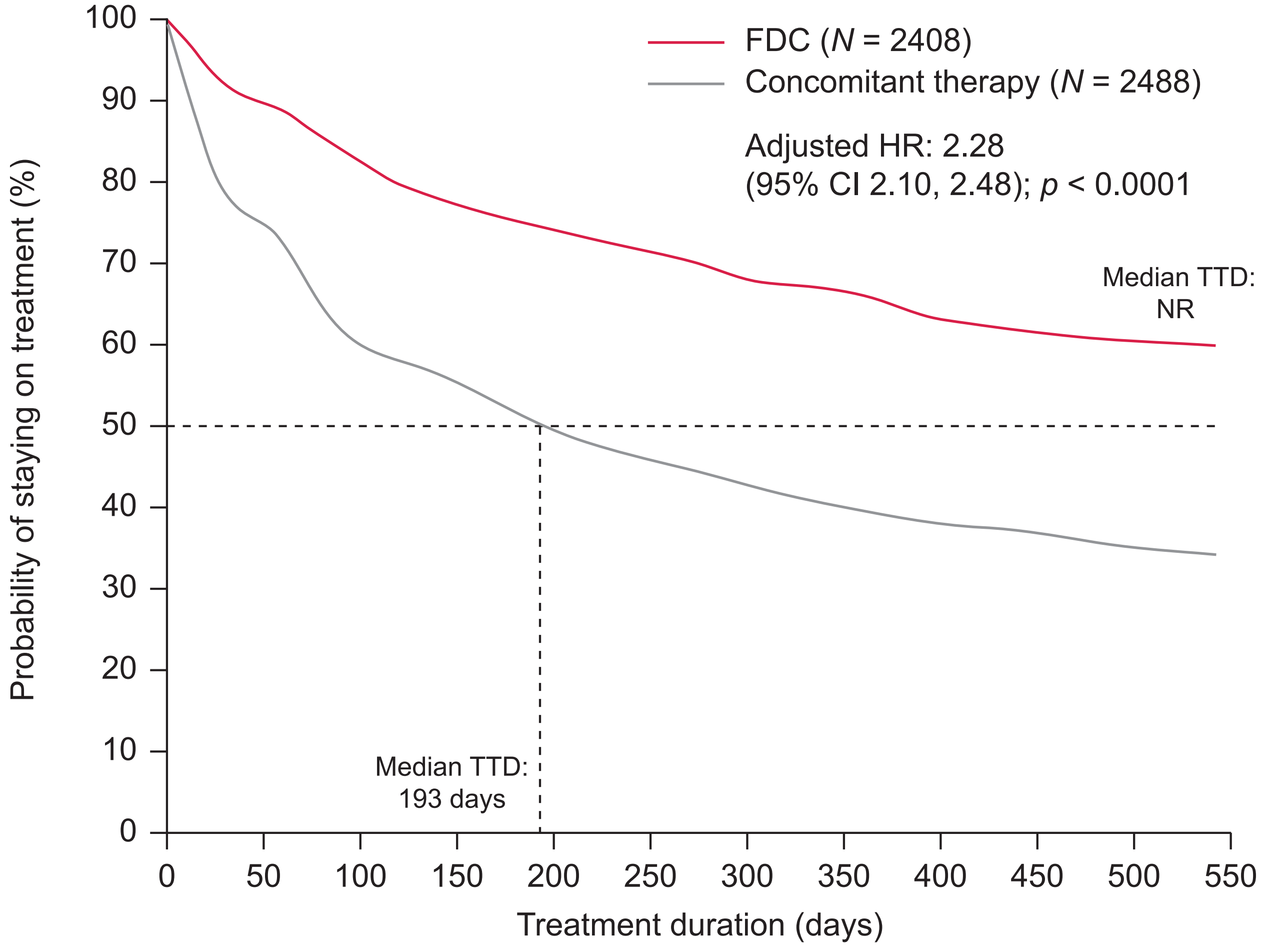

Figure S3. Median time to discontinuation in any FDC* compared with any concomitant therapy*. *α-blocker plus an antimuscarinic or 5-ARI. 5-ARI: 5α-reductase inhibitor; CI: confidence intervals; FDC: fixed-dose combination; HR: hazard ratio; NR: not reached; TTD: time to discontinuation
